# Spatiotemporal Phylogenetic Analysis and Molecular Characterisation of Infectious Bursal Disease Viruses Based on the VP2 Hyper-Variable Region

**DOI:** 10.1371/journal.pone.0065999

**Published:** 2013-06-21

**Authors:** Abdulahi Alfonso-Morales, Orlando Martínez-Pérez, Roser Dolz, Rosa Valle, Carmen L. Perera, Kateri Bertran, Maria T. Frías, Natàlia Majó, Llilianne Ganges, Lester J. Pérez

**Affiliations:** 1 Centro Nacional de Sanidad Agropecuaria (CENSA), La Habana, Cuba; 2 Universidad de las Ciencias Informáticas (UCI), La Habana, Cuba; 3 Centre de Recerca en Sanitat Animal (CReSA), Barcelona, Spain; 4 Departament de Sanitat i Anatomia Animals, Universitat Autònoma de Barcelona, Barcelona, Spain; University of Georgia, United States of America

## Abstract

**Background:**

Infectious bursal disease is a highly contagious and acute viral disease caused by the infectious bursal disease virus (IBDV); it affects all major poultry producing areas of the world. The current study was designed to rigorously measure the global phylogeographic dynamics of IBDV strains to gain insight into viral population expansion as well as the emergence, spread and pattern of the geographical structure of very virulent IBDV (vvIBDV) strains.

**Methodology/Principal Findings:**

Sequences of the hyper-variable region of the VP2 (HVR-VP2) gene from IBDV strains isolated from diverse geographic locations were obtained from the GenBank database; Cuban sequences were obtained in the current work. All sequences were analysed by Bayesian phylogeographic analysis, implemented in the Bayesian Evolutionary Analysis Sampling Trees (BEAST), Bayesian Tip-association Significance testing (BaTS) and Spatial Phylogenetic Reconstruction of Evolutionary Dynamics (SPREAD) software packages. Selection pressure on the HVR-VP2 was also assessed. The phylogeographic association-trait analysis showed that viruses sampled from individual countries tend to cluster together, suggesting a geographic pattern for IBDV strains. Spatial analysis from this study revealed that strains carrying sequences that were linked to increased virulence of IBDV appeared in Iran in 1981 and spread to Western Europe (Belgium) in 1987, Africa (Egypt) around 1990, East Asia (China and Japan) in 1993, the Caribbean Region (Cuba) by 1995 and South America (Brazil) around 2000. Selection pressure analysis showed that several codons in the HVR-VP2 region were under purifying selection.

**Conclusions/Significance:**

To our knowledge, this work is the first study applying the Bayesian phylogeographic reconstruction approach to analyse the emergence and spread of vvIBDV strains worldwide.

## Introduction

Infectious bursal disease (IBD) is a highly contagious and acute viral disease affecting all major poultry producing areas of the world [1]. This disease was initially identified in the 1960’s [2], and the most important lesions observed in affected animals are lymphoid tissue damage found in the bursa of Fabricius [3].

IBD is caused by the infectious bursal disease virus (IBDV), a non-enveloped virus belonging to the *Birnaviridae* family with a genome consisting of two segments of double-stranded RNA (segments A and B) [4]. Segment A encodes a precursor polyprotein in a major open reading frame (ORF) that is cleaved by auto-proteolysis to yield the mature VP2 (outer capsid), VP4 (protease), and VP3 (inner capsid) proteins [5]. The VP2 protein is largely recognised as the immunodominant antigen of IBDV [6].

This viral agent preferentially infects young, sexually immature 3–6-week-old chickens [7] and causes a variety of symptoms ranging from loss of feed efficiency to immunodeficiency, the latter of which leads to an increased susceptibility to other infectious diseases and a poor immune response to vaccines [3], [8].

Two different serotypes of IBDV that have been reported to date can be differentiated by virus neutralisation test [9]. All pathogenic isolates are serotype-1 strains, whereas serotype-2 strains neither cause disease nor protect against infection by serotype-1 strains [10], [11]. Based on pathogenicity and antigenicity characteristics, serotype 1 strains have been further classified as classical virulent IBDV (cvIBDV), very virulent IBDV (vvIBDV), antigenic variant IBDV (avIBDV) and attenuated IBDV (atIBDV) [12].

vvIBDV strains were first described in Belgium during the 1980’s in association with high mortality in young chickens [13], [14] and have been the source of significant economic losses in the poultry industry in many countries. The ability to cause high mortality in susceptible chickens is the primary defining quality of vvIBDV strains. To reduce the economic impact of vvIBDV strains, it is desirable to have a rapid, cost-efficient method to trace changes in virulence. Since in vivo studies are expensive, time-consuming and sometimes impossible, molecular techniques have been developed based on genotyping the hyper-variable region of VP2 (HVR-VP2) [15], [16], [17].

In Cuba, the first report of IBD was in 1982 [18]. Only sporadic outbreaks of the disease have been reported in the chicken flocks from 1982 to 1992 [19]. Although the Cuban flocks have been under a vaccination policy (reviewed in [20]) from 1993 to the present, a trend toward acute severe outbreaks of IBD in the chicken flocks has been documented [21]. An increase in severity of histopathological lesions of affected animals has also been observed [22]. However, genetic information on the IBDV field strains that circulate in Cuban flocks has remained unknown.

Bayesian inference methods have been recently developed that offer a unique opportunity to explore viral genotypic and phenotypic evolution in greater detail [23]. Phylogeographic approaches have helped uncover the impact that spatial epidemiological processes leave in the genomes of rapidly evolving viruses and have been used for tracking the spread of foot-and-mouth disease and human immunodeficiency virus type 1 [24], [25], [26].

The current study was designed to provide a rigorous measurement of the global phylogeographic dynamics of IBDV strains to gain insight into the expansion of viral populations as well as the emergence, spread and pattern of the geographical structure of vvIBDV strains. Analyses based on genealogy and phylogeography approaches were conducted to improve our understanding of the molecular epidemiology of IBDV and identify the possible origin of this agent in Cuba. We also assessed the possible selection pressure on the HVR-VP2 region resulting from the immune response elicited by host vaccination.

Here, a phylogeographic reconstruction was conducted for vvIBDV strains worldwide based on the HVR-VP2 region. This study revealed the expansion of strains carrying sequences linked to high virulence of IBDV from Iran in 1981 to Western Europe (Belgium) in 1987, Africa (Egypt) around 1990, East Asia (China and Japan) around 1993, the Caribbean Region (Cuba) around 1995 and finally South America (Brazil) around 2000.

## Materials and Methods

### Samples

#### Ethics statement

International standards for animal welfare were used for all animal samples collected, following the regulations for animal sampling of the Institute of Veterinary Medicine (IMV), Ministry of Agriculture (MINAGRI) of the Republic of Cuba. The protocol was approved by the Committee on the Ethics of the MINAGRI of the Republic of Cuba and all efforts were made to minimize suffering of the animals. Birds were euthanized using cervical dislocation to collect the samples. The samples were sent directly from the IMV to Animal Virology Laboratory at CENSA. The IMV is the official regulatory body of the Republic of Cuba; therefore additional permits were not required.

#### Collection, selection and processing of samples

Seventy-two bursa of Fabricius samples were initially included in this study. These samples were collected from 1992 to 2011 and came from commercial layer and broiler chicken flocks with suspected cases of IBD. Samples were collected from different geographic regions in Cuba and submitted to the Animal Virology Laboratory at CENSA for confirmatory IBDV diagnosis.

All bursa samples were split into two parts: one was frozen at −80°C to conduct molecular studies and the other was fixed in 10% formalin to conduct histopathologic examination following procedures previously described by González-Insua et al. [22]. Sixty-three bursa samples were confirmed as IBDV positive by immunohistochemistry tests following the assay described by Perera et al. [27]. Of these, 41 samples were selected for further molecular studies based on geographic region of origin and year of collection (Table 1). These selected bursal samples were printed on FTA® cards, suitable for the preservation of genetic material and adequate transportation [28]. Two atIBDV strains used as vaccines were also included in the current work: i) the commercial vaccine strain “Gumboro” (Labiofam, S.A., Cuba) (http://www.labiofam.cu/productos/vacuna-gumboro.html) that is currently used in vaccination programs by Cuban veterinary services (reviewed in [29]) and ii) the strain Lukert-I used as the vaccine “CT-Gumboral”, which was applied in Cuba between 1996–1999. These vaccine strains were also printed on FTA® cards and, together with the 41 FTA ® cards that contained field strains, were sent to Centre de Recerca en Sanitat Animal (CReSA) in Barcelona, Spain where molecular analyses were conducted.

**Table 1:** Sequences of Cuban IBDV strains used in the current study.

Year of collection	GenBank accession No.	Strain (ID used in tree)	Province
1992	HF547332	BL	La Habana
1995	HF547306	BF35Hab95	La Habana
1996	HF547333	117/96PiR96	Pinar del Río
1996	HF547305	29/96XHab96	La Habana
1996	HF547307	BF63Hab96	La Habana
1997	HF547334	BF6Gra97	Granma
1997	AJ238647*	Myga	La Habana
1997	HF547345	BF4Gra97	Granma
1997	HF547308	BF9Hab97	La Habana
1997	HF547310	BF3Hab97	La Habana
1997	HF547311	BF26Hab97	La Habana
1997	HF547312	BF8Hab97	La Habana
1997	HF547309	BF28Hab97	La Habana
1997	HF547344	BF7VCl97	Villa Clara
1997	HF547343	BF51Cam97	Camagüey
1997	HF547342	BF52Cam97	Camagüey
1997	HF547341	BF53CiA97	Ciego de Ávila
1998	HF547313	56/98Hab98	La Habana
1998	HF547314	61/98Hab98	La Habana
1998	HF547315	69/98Hab98	La Habana
1998	HF547340	BF67PiR98	Pinar del Río
1998	HF547316	BF70Hab98	La Habana
2000	HF547317	BF11Hab00	La Habana
2000	HF547339	47/00Cam00	Camagüey
2000	HF547318	94/00Hab00	La Habana
2000	HF547338	135/00Hol00	Holguín
2001	HF547319	11/01Hab01	La Habana
2002	HF547320	45/02Hab02	La Habana
2002	HF547321	BF12Hab02	La Habana
2004	HF547322	BF29Hab04	La Habana
2004	HF547323	BF31Hab04	La Habana
2006	HF547324	BF15Hab06	La Habana
2008	HF547337	BF14Cie08	Cienfuegos
2008	HF547325	BF16Hab08	La Habana
2009	HF547336	BF17Mat09	Matanzas
2009	HF547326	BF19Hab09	La Habana
2009	HF547329	BF18Hab09	La Habana
2010	HF547327	BF20Hab10	La Habana
2010	HF547328	BF22Hab10	La Habana
2011	HF547347	BF24Hab11	La Habana
2011	HF547330	BF25Hab11	La Habana
2011	HF547331	BF23Hab11	La Habana
2011	HF547335	Gumboro_labiofam”Vac/Cub11”	-
	HF547346	strain LukertI/Cuba	-

* Previously obtained and submitted at GenBank.

### NA Isolation from FTA® Cards, RT-PCR and Sequencing

RNA was extracted from all 43 FTA® card samples using the method described by Moscoso et al. [28] with modifications. Briefly, one gram from the spotted areas of the FTA® cards was cut by a sterile fork and placed in 1.5 ml microcentrifuge tubes. For each FTA® paper portion, 200 µL of nuclease-free water was added, vortexed and incubated for 10 min at room temperature. The final suspensions were centrifuged at 7500 g for 5 min at 4°C. RNA was then extracted from the 150 µL of recovered supernatant using the Nucleospin RNA virus kit (Macherey-Nagel, Düren, Germany) following the manufacturer’s instructions. RNA was eluted in a final volume of 60 µL of nuclease-free water.

The RNA extracted from FTA® cards was used to amplify the complete HVR-VP2 using the primer pair GUM-F (5′-ACAGGCCCAGAGTCTACA-3′)/GUM-R (5′-AYCCTGTTGCCACTCTTTC-3′) and RT-PCR conditions previously described by Dolz et al. [30]. Amplification products were visualised by electrophoresis on 1.8% agarose gels stained with ethidium bromide and cleaned with the QIAquick PCR purification kit (Qiagen Inc., Valencia, CA) following the manufacturer’s instructions.

The resulting products were submitted to bi-directional DNA sequencing using a BigDye Terminator v3.1 cycle sequencing kit following the manufacturer’s instructions (Applied Biosystems, Stockholm, Sweden). Sequencing products were read on an ABI PRISM-3100 Genetic Analyzer (Applied Biosystems, Stockholm, Sweden). The sense and antisense sequences obtained from each amplicon were assembled, and a consensus sequence for each gene was generated using the ChromasPro V1.5 program (Technelysium Pvt. Ltd., 2009). Nucleotide BLAST analysis (http://www.ncbi.nlm.nih.gov/blast/Blast.cgi) was initially used to verify the identity of each fragment sequence obtained. Sequences were submitted to the GenBank database under accession numbers HF547305–HF547347.

### Selection of the Sequence Dataset and Multiple Sequence Alignment

To establish phylogenetic relationships and classification the Cuban field strains included in the present work (Table 1), Cuban sequences were initially aligned along with 67 HVR-VP2 sequences available in the GenBank database (Table S1). Thus, an initial dataset of 111 sequences that included different geographic origins and different classification was built. To estimate rates of nucleotide substitution per site, per year and the time to the most recent common ancestors (tMRCAs) of specific groups, only sequences with a known year of collection were included. The Cuban vaccine strains were also removed. Thus, 25 sequences included in the first analysis were removed (Table S1, the sequences removed are denoted by a symbol (‡)). In all cases, alignments of the dataset of sequences were performed using the algorithm ClustalW method included in the program BioEdit Sequence Alignment Editor [31].

### Phylogenetic Analysis

To remove sequences with a possible recombination event, searches for recombinant sequences and crossover regions were performed from alignment of the 111 sequence dataset using Geneconv [32], RDP [33], MaxChi [34], [35], Chimera [35], BootScan [36], SiScan [37], 3Seq [38] and LARD [39], all implemented in RDP3 Beta 4.1 [40]. Programs were executed with modified parameter settings according to the guidelines in the RDP3 manual for the analysis of divergent sequences (available upon request). Recombinant sequences were tested with the highest acceptable p value of 0.05, and Bonferroni’s multiple comparison correction was used. Analyses were conducted twice to ensure the repeatability of the results.

ModelTest V.3.0.6 [41] was used to estimate the best-fit model using the Akaike information criterion (AIC). The best-fit model was selected and used for phylogenetic analysis. Phylogenetic relationships among IBDV strains based on HVR-VP2 sequences were analysed using Bayesian inference (BI) and maximum likelihood (ML) methodologies. Bayesian inference analyses were performed with the MrBayes 3.1 software [42], [43]. MCMC searches were run with four chains for 10 million generations and sampling of the Markov chain every 100 generations. At the end of the run, the convergence of the chains was inspected through the average standard deviation of split frequencies; the first 25% of the trees were discarded. After discarding the burn-in, the four MCMC chains were combined and summarised on a majority-rule consensus tree. The convergence was again assessed on the basis of the effective sampling size (ESS) using Tracer software version 1.4 (http://tree.bio.ed.ac.uk/software/tracer/). Only log-likelihoods with ESS values of >250 were accepted. A tree with clade credibility was constructed using the posterior probability distribution. The tree was rooted using the sequences of IBDV serotype 2 accessed from the GenBank database (accession number M66722).

ML trees were computed using PHYML v3.0 [44], and confidence levels were estimated by 1000 bootstrap replicates. The topologies were tested by the Kishino and Hasegawa test (K–H) [45] and the Shimodaira–Hasegawa test (S–H) [46], which computed the log-likelihood per site for each tree and compared the total log-likelihood for each proposed topology using the PAMLv4.3 program [47]. Ten thousand replicates were performed using the K–H and S–H topologies tests by re-sampling the estimated log-likelihoods for each site (RELL model) [48]. Finally, the selected tree was visualised by FigTree v1.1.2 [49].

### Substitution Rates, Time-scale of Evolutionary History and Phylodynamic Analyses

The dataset of 86 previously selected sequences (see section 2.3) was used to generate the BEAST input file by BEAUti within the BEAST package v1.7.4 [50] (freely available at http://beast.bio.ed.ac.uk). Rates of nucleotide substitution per site and per year and the tMRCA were estimated by a Bayesian Markov chain Monte Carlo (MCMC) approach. Model selection was performed by estimating model marginal log-likelihood through the AICM following the method described by Baele et al. [51]. The estimation of model marginal log-likelihood through the AICM for the twelve coalescent demographic models included parametric models (constant population size, exponential and logistic growth) and nonparametric models (Bayesian skyline plot, BSP) with strict (SC), uncorrelated lognormal distribution (UCDL) and uncorrelated exponential distribution (UCED) relaxed molecular clocks were calculated (Table S2). Rates of nucleotide substitution per site and per year and the tMRCA were also estimated only for Cuban sequences. A Bayesian skyline plot was also created from a sub-dataset of the HVR-VP2 sequence, which only included the Cuban sequences, to infer the population dynamics of Cuban viruses as measured by differences in the levels of relative genetic diversity (Neτ) over time.

In all cases, the MCMC chains were running for 100 million generations to obtain an ESS >250. The first 10% of trees were discarded as “burn-in” as recommended by the BEAST package manual [52] (freely available at http://beast.bio.ed.ac.uk). Convergence was assessed by estimating the ESS after a 10% burn-in using Tracer software version 1.5 (http://tree.bio.ed.ac.uk/software/tracer/). The tree with maximum log clade credibility was selected and visualised by FigTree v1.1.2 [49].

### Phylogeny-trait Association and Phylogeographic Analysis

The association between phylogeny and the pattern of the geographical structure of IBDV was assessed using the software BaTS [53]. In a first approach, the trait of geographic region for the 111 sequence dataset was assessed by removing only the vaccine strains from this dataset. In a second analysis, the association between the different phylogenetic relationships of the field strains present in Cuba and Cuban provinces was performed by analysing only the Cuban sequences (vaccine strains were not included). Values of the association index (AI), parsimony score (PS) statistics and the level of clustering in individual locations using the monophyletic clade (MC) size statistic were all calculated based on the posterior samples of trees produced by MrBayes 3.1 using the BaTS program. The null distribution for each statistic was estimated with 1,000 replicates of state randomisation.

A discrete phylogeographic analysis (DPA) was conducted using a standard continuous-time Markov chain (CTMC) model with Bayesian stochastic search variable selection (BSSVS) to model the geographic transmission of vvIBDV from the 65 sequence dataset [54]. The MCMC analyses were performed in two independent runs. After burn-in, the two runs were combined to summarise the results. The resulting maximum clade credibility (MCC) phylogenetic trees were obtained by TreeAnnotator. A second DPA was performed to model the spatial diffusion of IBDV between Cuban provinces using the same conditions described above and analysing only the Cuban sequences.

The results of both DPA were summarised using the SPREAD software [55]; keyhole markup language (KML) files were generated to identify the major routes of geographic diffusion. The Bayes factor (BF) test was used to select the most probable routes of transmission. The resulting KML files from SPREAD with a non-zero expectancy that showed a BF>5 for the global analysis and a BF>3 for the Cuban province diffusion analysis were visualised by Google Earth (available at: http://earth.google.com).

### Analysis of Selection Pressures

The existence of selection pressure on HVR-VP2 in Cuban IBDV sequences was assessed by plotting the amino acid sequence entropy calculated by the DAMBE program [56]versus the difference between nonsynonymous (dN) and synonymous (dS) rates calculated by the SNAP web utility (http://hiv-web.lanl.gov/content/hiv-db/SNAP/WEBSNAP/SNAP.html) based on the method of Nei and Gojobori [57]. The selection pressures at individual codons were estimated using the single likelihood ancestor counting (SLAC), fixed effects likelihood (FEL) methods available at the Datamonkey online version of the HyPhy package [58], [59]. All analyses utilised the TN93+G model of nucleotide substitution and employed input maximum likelihood phylogenetic trees.

Finally, to detect positive selection acting on a particular lineage as well as sites within the HVR-VP2, several models available in the CODEML module of PAML 4.3 software package [60] were used. Different values of the non-synonymous/synonymous dN/dS rate ratio (x parameter) were considered according to the user guide [60]. To avoid false positives, the models used to detect sites under positive pressure were contrasted with models used to detect neutral selection [61]; only cases where the likelihood ratio test (LRT) result was significant were considered. The Bayes empirical Bayes (BEB) calculation of posterior probabilities for site classes was used to calculate the probabilities of sites under positive selection [47].

Chimera software package (http://www.cgl.ucsf.edu/chimera) and Swiss-PdbViewer (http://spdbv.vital-it.ch/) were used to visualise the 3D crystal structure of VP2.

## Results

### Phylogenetic Relationships and Sequence Analyses

**Figure 1:**
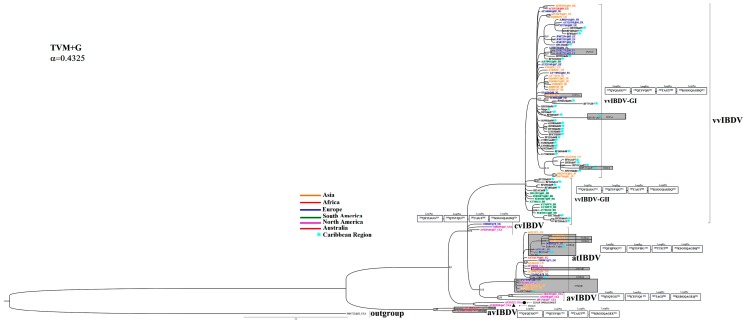
Phylogenetic tree of IBDV based on HVR-VP2. The model used for phylogenetic inference is shown at the top left. The Cuban IBDV sequences are denoted with black letters and the names of the strains were used as identification (ID) in the tree. For the remaining sequences, an ID for the tree was used (see Table S1). For geographic-trait association, different colours related to the origin of the strain were used and denoted (strains from Asia in orange, Africa in red, Europe in blue, South America in green, North America in pink, Australia in brown and the Caribbean Region with a turquoise star). The pattern of the HVR-VP2 loop structures P_BC_, P_DE_, P_FG_, and P_HI_ linked to changes in IBDV virulence and escape from vaccination by Coulibaly et al. (2005) are shown as rectangles. Those strains that showed changes in this pattern are highlighted using a grey rectangle; the amino acid substitution is also indicated.

The phylogenetic relationships among the IBDV strains were reconstructed based on HVR-VP2 sequences using ML and BI analyses. Both algorithms yielded congruent results with the same topology, which was supported by moderate to high confidence values given by the bootstrap percentage and the posterior probability (data not shown). The best Bayesian tree was obtained by the S-H test, but the support obtained for this tree was not significantly different from the ML tree (data not shown). The Bayesian tree that estimated the phylogenetic relationships between the Cuban IBDV sequences and those from other geographical regions are shown in Fig. 1.

Three Cuban IBDV field strains (BL, BF23Hab11 and BF6Gra97) were grouped in the defined cluster corresponding to the atIBDV strains together with the vaccine strains applied in Cuba (Fig. 1). The remaining Cuban IBDV field strains were grouped in the defined cluster corresponding to the vvIBDV strains. However, a clear division into two subgroups was observed among the vvIBDV strains included in the current work (vvIBDV-GI and vvIBDV-GII) (Fig. 1). Thus, the Cuban IBDV strains BF35Hab95, 29/96XHab96, 117/96PiR96, BF63Hab96, Myga97, BF26Hab97, BF28Hab97, BF3Hab97, BF4Gra97, BF51Cam97, BF52Cam97, BF53CiA97, BF7VCl97, BF8Hab97, BF9Hab97, BF67PiR98, BF70Hab98, 56/98Hab98, 61/98Hab98, 69/98Hab98, 47/00Cam00, 94/00Hab00, BF11Hab00, 135/00Hol00, 11/01Hab01, 45/02Hab02, BF12Hab02, BF29Hab04, BF31Hab04, BF29Hab04, BF31Hab04 and BF16Hab08 collected between the years 1995–2008 (Table 1) were located in the vvIBDV-GI cluster, whereas the Cuban IBDV strains BF14Cie08, BF17Mat09, BF18Hab09, BF19Hab09, BF20Hab10, BF22Hab10, BF24Hab11, BF25Hab11, collected between 2008–2011 (Table 1), were located in the vvIBDV-GII cluster (Fig. 1).

Comparisons of nucleotide and deduced amino acid sequences were carried out among the Cuban IBDV field strain sequences obtained in the current study. Nucleotide sequence identities among the HVR-VP2 sequences of the 42 Cuban IBDV field strains ranged from 91.6–100% and those of the deduced amino acids ranged from 91.3–100%.

The region of the four loop structures (P_BC_, P_DE_, P_FG_, and P_HI_) of the HVR-VP2 has been linked to a change in IBDV virulence and escape from vaccination [62].The lineages on the tree (Fig. 1) can be correlated with specific loop structure mutations. Based on the pattern of the four loops for each lineage, comparisons of amino acid substitutions were performed among all sequences included in the present work. Amino acid changes in the pattern described for vvIBDV were only found in the strains BF53CiA97 and BF52Cam97, which showed changes of **222PxA** and **286IxT** in loops P_BC_ and P_FG_, respectively (Fig. 1). Amino acid changes in the pattern of atIBDV were found in the three Cuban field strains. A replacement of **249RxQ** was found in the strains BL and BF6Gra97, while a replacement of **253QxH** was found in the strain BF23Hab11, all located in loop P_DE_ (Fig. 1).

For Cuban sequences, amino acid changes in other regions of the HRV-VP2 (outside of the loop structures) were also compared. Two different groups of sequences were obtained to perform a better comparison by taking into account the IBDV strain classification based on the phylogenetic tree obtained (Fig. 1). The first group was formed by sequences of strains classified as atIBDV (Fig. 1) and the vaccine strain “Gumboro” was used as a reference. Only strain BF6Gra97 showed a replacement of **242VxA**. The second group was formed by sequences of strains classified as vvIBDV (Fig. 1) along with strain BF35Hab95, which was used as a reference. In this group, the strains 29/96XHab96 and BF7VCl97 showed a replacement of **212NxD**. Strain BF7VCl97 also showed the replacements **337IxT**, **340RxG** and **341TxN**. In strain BF52Cam97, outside of the loop structures, the replacement **334VxA** was found, while strain BF53CiA97 showed the replacements **211DxA**, **279NxD**, **299NxS** and **330RxA**. Interestingly, the replacement **299NxS** was also found in the strains BF14Cie08, BF16Hab08, BF17Mat09, BF19Hab09, BF18Hab09, BF20Hab10, BF22Hab10, BF24Hab11 and BF25Hab11, which are the most recent strains included in the group vvIBDV-GII (Fig. 1).

### Time-measured Phylodynamic Analyses

AICM analysis based on an analogue of AIC [51] showed a skyline coalescent plot and an exponential, uncorrelated clock best fitted to our data (Table S2).

**Figure 2:**
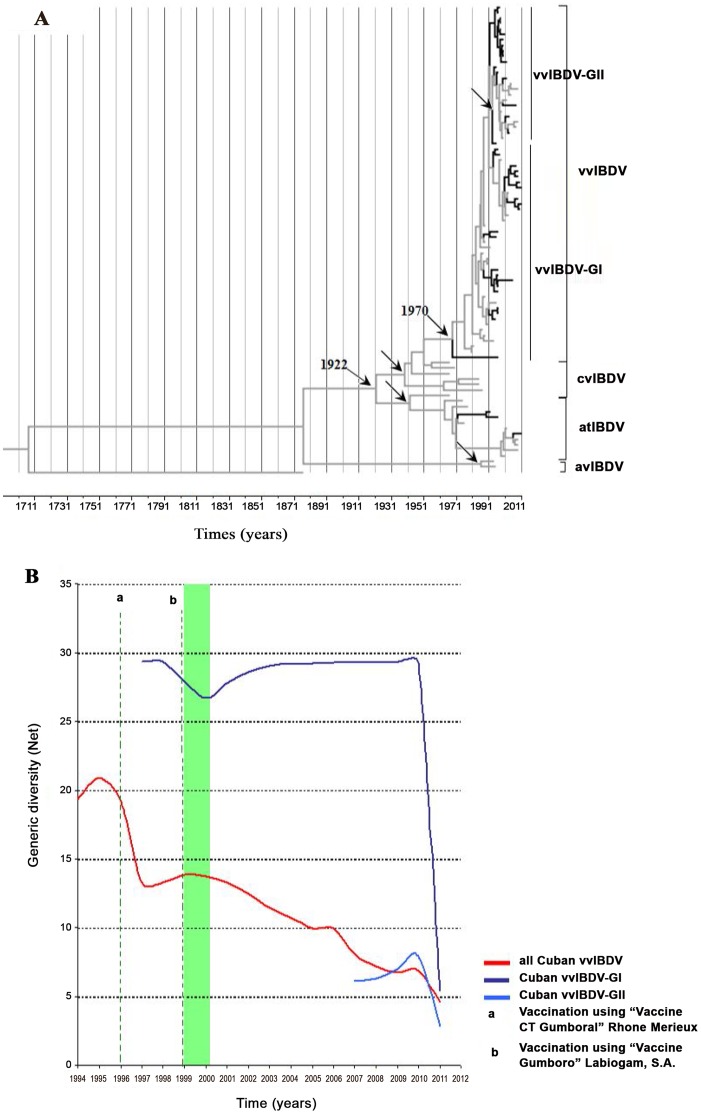
Phylodynamics of IBDV. The left panel A) describes a maximum clade credibility (MCC) analysis performed using BEAST (Drummond et al., 2012), with the evolutionary history of the HVR-VP2 sequences dataset shown. The branches belonging to Cuban sequences were highlighted in black. The internal nodes of cvIBDV, atIBDV, avIBDV and vvIBDV were denoted using black arrows and the most probable year for the MRCA of vvIBDV was also denoted. The right panel B) shows a Bayesian skyline plot (BSP) representing the relative genetic diversity of circulating Cuban vvIBDV over time. In addition, a BSP to differentiate the genetic diversity of the two different groups of vvIBDV found circulating in Cuba was also shown. Different colours were used to distinguish each population (red line: all Cuban vvIBDV strains, blue line: Cuban vvIBDV included in group I and sky-blue line: Cuban vvIBDV included in group II). For clarity, only the mean population sizes over time were plotted. The starting period for the vaccination program supported by serological test was highlighted by a green shadow.

The estimated mean (95% HPD) for the substitution rate of IBDV was 8.63×10^−4^ (4.14×10^−4^–1.41×10^−3^) substitutions/site/year. The date Bayesian phylogenetic tree obtained for the global IBDV strains was characterised by a clear temporal structure; the oldest samples tended to fall closest to the root of the tree, while the most recent samples were located at the most distal tips. The mean tMRCA for the diversification of IBDV serotype 1 to the different linages cvIBDV, atIBDV and vvIBDV was located at approximately 1922 95% HPD from 1869 to 1960), while the mean of tMRCA for the vvIBDV strains was 1970 (95% HPD from 1956 to 1985) (Fig. 2A).

The estimated mean (95% HPD) of the substitution rate for Cuban IBDV strains was 1.23×10^−3^ (5.98×10^−4^–1.9×10^−3^) substitutions/site/year, and the mean of the tMRCA for the Cuban IBDV strains was 1977 (95% HPD from 1952–1992). In addition, the substitution rate was 1.94×10^−3^ (1.04×10^−3^−2.95×10^−3^) substitutions/site/year for the Cuban vvIBDV strains, and the mean of the tMRCA was 1991 (95% HPD from 1984–1995) (Table 2). The estimated substitution rates and the tMRCA for the different groups of Cuban vvIBDV strains (GI and GII) are also shown in Table 2.

**Table 2:** Estimated substitution rates and tMRCA for the Cuban IBDV strains.

Dataset of Cuban IBDV strains	Rate (substitution/site/year) (HPD95%)	tMRCA (year) (HPD95%)
Whole population	1.23×10^−3^(5.98×10^−4^–1.9×10^−3^)	1977 (1952–1992)
vvIBDV	1.94×10^−3^(1.04×10^−3^–2.95×10^−3^)	1991 (1984–1995)
vvIBDV-GI	1.42×10^−3^(6.25×10^−4^–2.29×10^−3^)	1994 (1986–1998)
vvIBDV-GII	5.25×10^−3^(2.11×10^−3^–4.94×10^−3^)	2007 (2008–2006)

Demographic inference using the BSP model is summarised in Fig. 2B, which essentially plots Neτ as a function of time. Neτ can be considered a measure of relative genetic diversity that reflects the number of effective infections established by the virus. Therefore, the BSP for the whole vvIBDV Cuban population showed peaks for Neτ during the second half of 1994 until 1996, indicating an epidemic behaviour of the virus (Fig. 2B). In contrast, a decrease in Neτ in 1997 for the whole Cuban population of vvIBDV was observed, with a quick inflection suggesting a new increase in diversity of the population. However, a discontinuous decrease of genetic diversity was observed since 1999, with two epidemic peaks of Neτ in the years 2006 and 2010. It is important to emphasise that in 1999, a new vaccination program against IBDV supported by serology test was applied to the Cuban flocks.

On the other hand, a decrease in Neτ in 1998 for the Cuban vvIBDV-GI subpopulation was observed, with a trend towards a constant size or stabilisation of this subpopulation between the years 2000–2011. This finding provided evidence of endemic circulation of this subpopulation (Fig. 2B). Thus, the increase in Neτ of the whole Cuban population of vvIBDV during 2006 and 2010 seem to be related to two events: the first one is the emergence of the Cuban vvIBDV-GII subpopulation from 2006–2007, and the second is an increase in Neτ of the latter subpopulation (Fig. 2B). Finally, Neτ dramatically dropped from the second half of 2010 to 2011.

### Phylogeographic Analysis

#### Overall patterns of geographic structure

The global trait association (AI and PS) tests of phylogeographic structure rejected the null hypothesis of no association between sampling location and phylogeny at all spatial levels tested for the vvIBDV strains included in the present study (Table 3). Thus, the HVR-VP2 sequences possess some geographic structure. The use of index ratios of the observed values to those expected under panmixis (where 0 indicates complete population subdivision and 1 suggests random mixing [panmixis]) allows the strength of the association between geography and phylogeny to be further characterised. Hence, the AI of 0.40 (0.31–0.53B CI) suggests that the evolution of vvIBDV is not homogeneous but rather presents a geographic structure. This characteristic was more evident when the MC statistic was shown (Table 3). The population subdivision was significant for most localities, although samples from the Africa and South America showed evidence of gene flow (Table 3).

**Table 3:** Phylogeny-trait association tests of the phylogeographic structure of vvIBDV using BaTS.

Analysis	Statistic	IR(CI95%)	Observed mean(CI95%)	Expected mean (CI95%)	Significance	
**Global**	AI	**0,40 (0,31–0,53)**	**3,1 (2,6–3,68)**	**7,7 (6,9–8,4)**	<0,001	
	PS	**0,63 (0,57–0,70)**	**37,0 (39,0–35,0)**	**58,7 (55,3–61,4)**	<0,001	
	MC (Asia)	ND	**6,0 (6,0–6,0)**	**1,9 (1,3–2,5)**	<0,001	
	MC (Australia)	ND	**2,0 (2,0–2,0)**	**1,0 (1,0–1,0)**	<0,001	
	MC (Africa)	ND	1,0 (1,0–1,0)	1,0 (1,0–1,0)	1	
	MC (Europe)	ND	**3,0 (3,0–3,0)**	**1,7 (1,1–2,3)**	0,01	
	MC (South America)	ND	1,0 (1,0–1,0)	1 (1,0–1,0)	1	
	MC (North America)	ND	**2,1 (1,0–4,0)**	**1,2**	0,03	
	MC (Caribbean Region)	ND	**3,0 (3,0–3,0)**	**1,2**	<0,001	
**Cuban provinces**	AI	0,9 (0,5–1,4)	1,8 (1,3–2,2)	2,0 (1,5–2,4)	0,2	
	PS	0,98 (0,91–1,1)	11,6 (11,0–12,0)	11,8 (11,0–12,0)	1,0	
	MC (La Habana)	ND	3,8 (3,3–4,0)	4,7 (3,1–8,1)	0,7	
	MC (Granma)	ND	1,0 (1,0–1,0)	1,0 (1,0–1,0)	1,0	
	MC (Villa Clara)	ND	1,0 (1,0–1,0)	1,0 (1,0–1,0)	1,0	
	MC (Camaguey)	ND	1,2 (1,0–2,0)	1,1 (1,0–1,2)	1,0	
	MC (Ciego de Avila)	ND	1,0 (1,0–1,0)	1,0 (1,0–1,0)	1,0	
	MC (Pinar del Rio)	ND	1,2 (1,0–2,0)	1,0 (1,0–1,0)	1,0	
	MC (Holguin)	ND	1,0 (1,0–1,0)	1,0 (1,0–1,0)	1,0	
	MC (Cienfuegos)	ND	1,0 (1,0–1,0)	1,0 (1,0–1,0)	1,0	
	MC (Matanzas)	ND	1,0 (1,0–1,0)	1,0 (1,0–1,0)	1,0	

In contrast, phylogeny-trait association tests based on AI and PS yielded no significant associations of the relationship among the field strains in Cuba and the Cuban provinces (Table 3). This result further indicates that the vvIBDV evolution is homogeneous throughout Cuba.

#### Inference of routes of vvIBDV spread and local spreading

**Figure 3:**
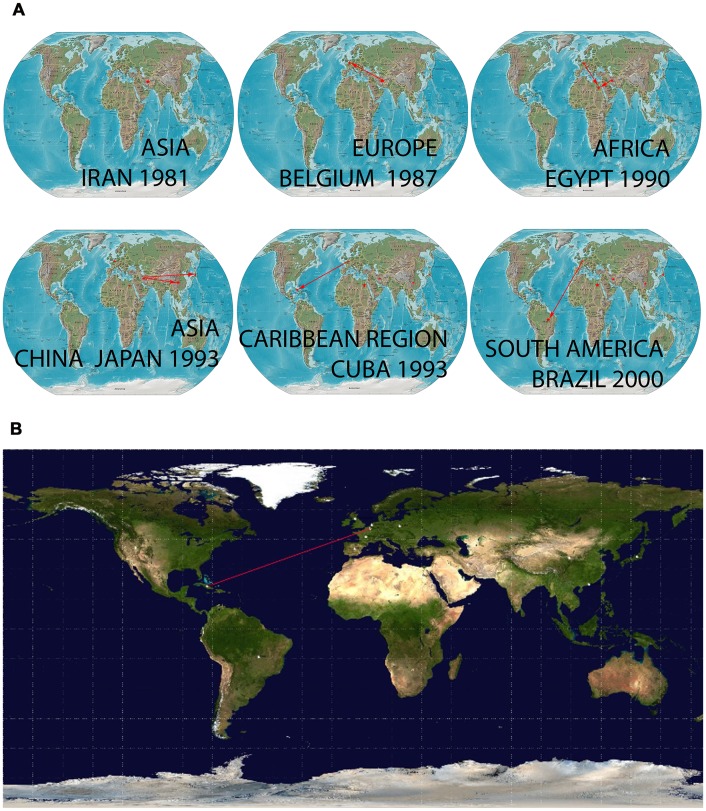
Temporal dynamics of spatial vvIBDV diffusion. A) Temporal dynamics of spatial vvIBDV diffusion globally; only rates supported by a BF of >5 were considered significant. The map was reconstructed using the Gateway to Astronaut Photography of Earth (public domain: http://eol.jsc.nasa.gov/sseop/clickmap/), this panel is similar but not identical to the original image, and is therefore for illustrative purposes only (whole video included as Video S1). B) Most probable route for the arrival of vvIBDV to Cuba with a BF of >20 was considered significant. The map was directly taken from the output file of the spread software.

Phylogeographic reconstruction was able to identify a single location for the root of the tree of the global dataset of vvIBDV sequences with posterior probabilities for state sp = 0.31 for the locality of Iran. The virus then spread following two distinct routes: i) to Europe arriving at Belgium and the Netherlands in 1987 and 1988, respectively and ii) from North East Asia arriving to China and Japan around 1992 (Fig. 3A and whole video included as Video S1). vvIBDV strains arrived in the Caribbean Region around 1995 and in South America around the year 2000 (Fig. 3A and whole video included as Video S1). Viral strains from the United Kingdom, the Netherlands and Belgium were the most probable sources for the introduction of vvIBDV strains to Cuba (Video S1, BF>5), even though only the route from Belgium showed a BF>20 (Fig. 3B).

**Figure 4:**
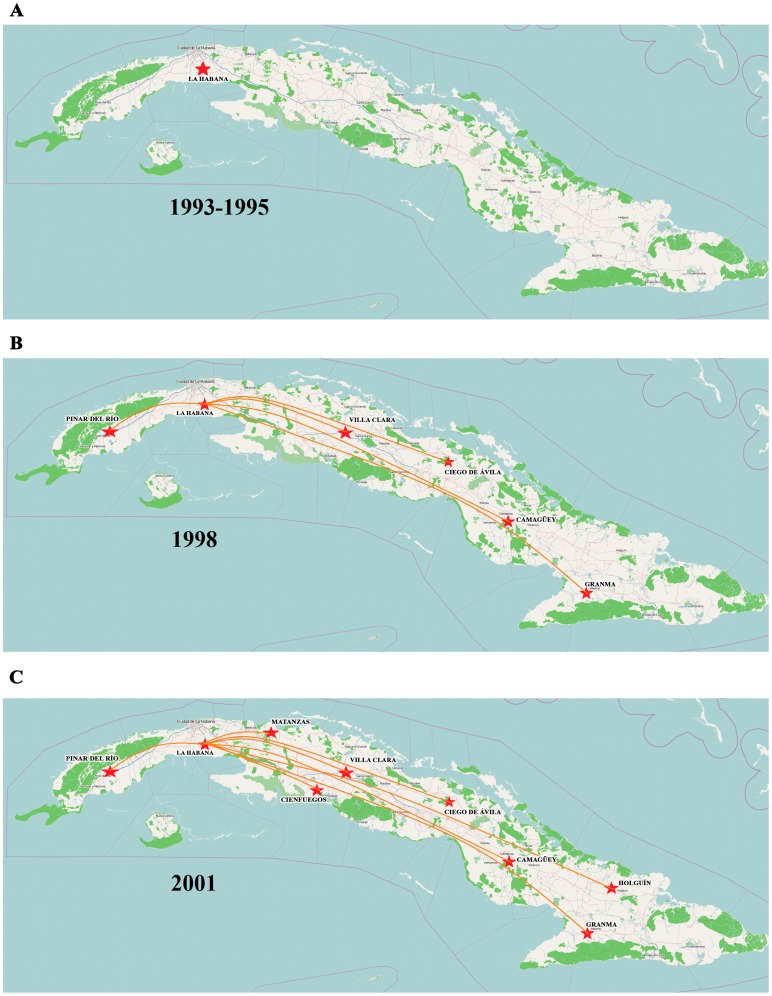
Inferred routes of spread of vvIBDV in Cuba. A) For the years of 1993–1995, B) 1998 and C) 2001. Only rates supported by a BF of >3 were considered significant. The maps were reconstructed using OpenStreetMap (http://www.openstreetmap.org/), this figure is similar but not identical to the original image, and is therefore for illustrative purposes only.

Once vvIBDV arrived in Cuba, the virus quickly spread from the western region of the country to the east in only three years (Fig. 4A & B), reaching complete dissemination throughout the country by 2001 (Fig. 4C). The phylogeographic analysis placed the Havana region as the most probable entry point into Cuba; from there, the virus spread by different routes (BF>3) to the rest of the country.

### Selection Pressures

**Figure 5:**
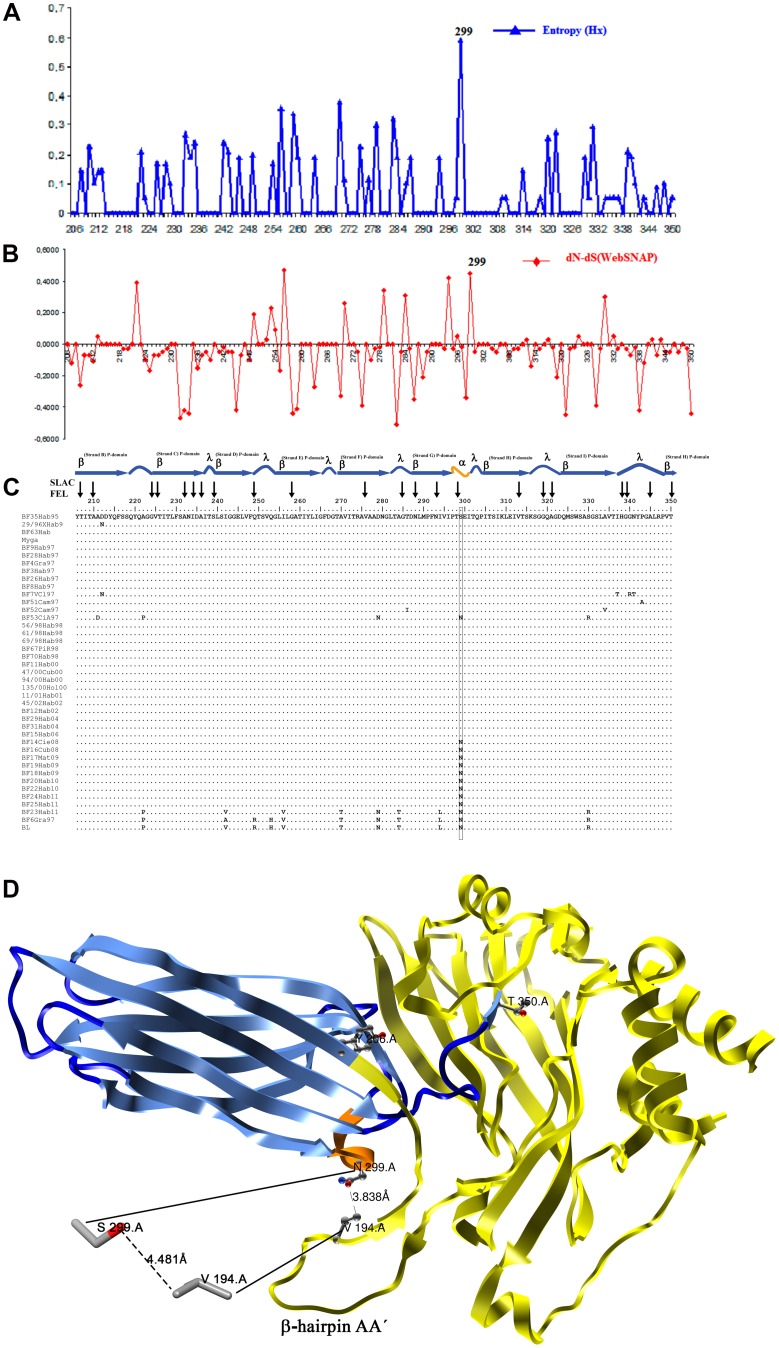
Analysis of amino acids of the HVR-VP2 region of Cuban IBDV strains. **Panel (A)** Amino acid entropy rates plot, x-axis: amino acid position; y-axis: entropy. The measure of entropy (Hi) for each position of amino acid was obtained using DAMBE software. The position with the highest value of entropy is indicated. **Panel (B)** Difference between non-synonymous and synonymous rates (dN-dS). Codon-specific nonsynonymous (dN) and synonymous (dS) substitution rates were obtained via a website (WEBSNAP); position 299 was denoted. **Panel (C)** Amino acid sequence alignment of the Cuban field strains of IBDV. Those residues under purifying selection as detected by SLAC and FEL analyses are indicated with black arrows. The secondary structure that belongs to each residue of the alignment is also denoted: β indicates β-sheets (in sky blue), λ indicates loops (in dark blue) and α indicates α-helices (in orange). The residue at position 299 is highlighted using a black rectangle. **Panel (D)** X-ray crystal structures of a monomer of VP2, crystal structure *2DF7* was downloaded from Protein Data Bank; only the monomer A was used for clarity. Chimera software v1.6.2 was used for visualisation. Residues Y206 and T350, respectively indicating the beginning and end of HVR, are shown. The HVR is annotated using the same colours as panel (C): β-sheets in sky blue, loops in dark blue and α-helices in orange. The remaining domains (S and B) are denoted in yellow. The β-hairpin AA′ involved in the stabilisation of the trimer conformation of VP2 (Coulibaly et al., 2005) is indicated. The distance between residues 299 and 194 is shown.

No residues under positive selection pressure were detected in the HVR-VP2 by the PAML software with significant values (p<0.05). In addition, the PAML software yielded values of (ω = dN/dS) very close to one for models M2 and M8, suggesting that most mutations found were under neutral selection (Table S3). In contrast, the SLAC and FEL methods yielded values for 22 codons under negative selection pressure (purifying selection) with p<0.05 (Fig. 5 and Table S4). The highest value of variability, as indicated by the entropy for each codon position, was found for codon 299. This position also showed the highest value of dN-dS by WebSNAP-utility along with position 256 (Fig. 5). Therefore, these results suggest that changes in the position 299 over time could be the most advantageous mutations and considered as antigenic drift. The distance between the residue at position 299 and the valine (V) at position 194, located on the β-hairpin AA′ structure of VP2, was also calculated. This estimate showed that the distance when the N residue was located at this position was lower (3.833 Å) than when the S residue was found at this position (4.481 Å) (Fig. 5).

## Discussion

In the past, efforts have been made to infer the dispersal of IBDV and to elucidate the diffusion patterns of the virus over decades [63], [64], [65]. However, only the recent introduction by Lemey et al. [54] of a Bayesian phylogeographic inference framework allowed for the incorporation of spatial and temporal dynamics of viral lineages for inference, visualisation, and phylogeographic hypothesis testing. To our knowledge, the current study is the first applying the Bayesian phylogeographic reconstruction approach to gain insight into the emergence and spread of vvIBDV strains worldwide. Furthermore, the genetic diversity and spatiotemporal dynamics of the Cuban IBDV strains as representatives of the Caribbean Region, framed on a global stage, were also analysed.

The expansion of vvIBDV in the late 1980s dramatically changed the epidemiology of the disease. Thereafter, several studies were conducted to obtain a better understanding of the evolution and spread of vvIBDV worldwide [15], [63], [64], [65] Although some studies have included segment B of the viral genome to obtain more accurate inference of IBDV phenotypes [63], [65], partial VP2 sequences have been widely used as a reliable approach [15], [16], [17], [30], [64].

Based on the HVR-VP2 sequences, the Cuban IBDV strains analysed in the current work were classified as atIBDV (n = 3) and vvIBDV (n = 38). The most likely date of first introduction of the virus to the country was 1977 (1952–1992), which was concordant with the first report of the presence of IBDV in Cuba in 1982 [18]. The date for the emergence of vvIBDV in Cuba was estimated to be 1991 (1984–1995). This event appears related to an increase in the number and severity of field outbreaks from 1993 to the present [19], [21].

We estimated the emergence for vvIBDV in the global scenario around 1970. This date is in agreement with the range of years proposed by Hon et al. [63], who determined the emergence of tMRCA for the VP2 sequences associated with highly virulent strains of IBDV among 1950–1973. In the present work, the expansion of strains carrying HVR-VP2 sequences linked to high virulence of IBDV was fixed starting from Iran in 1981 (see Video S1). IBDV was first reported in Iran in 1981 and was associated with low mortality rates in a broiler farm [66]. Our analysis suggested that the Iranian IBDV isolates RT275/81 and RT75D/82 (GenBank acc. No. DQ630451 and DQ630455, respectively) were the ancestral isolates of the virus that subsequently spread to Belgium in 1987.

Hon et al. [63] hypothesised that the expansion of highly virulent strains of IBDV occurred around 1981. This process of expansion was linked to the reassortment of the genome (segment B) of IBDV with a mutant VP2 background, which caused a sudden increase in virulence [63]. It has also been suggested that several migratory and sedentary avian species, such as cattle egrets, pigeons, carrion crows and waterfowl species (bean goose, white-fronted goose, and mallard duck), may also be carriers or reservoirs of IBDV [17], (reviewed in [63]). Considering that the three most important global flyways for several migratory avian species cross Iran on the way to Europe and East Asia (see Fig. S1), we can hypothesise that the viral strains that caused the 1981 outbreak in Iran could have provided the background for the VP2 sequences of the vvIBDV strains. The virus could have been transported to Belgium by the migration of avian species, in which segment B reassortment events may have taken place, triggering the increase in virulence.

Unfortunately, to our knowledge, very few studies have been conducted so far to determine whether wild birds can become infected with IBDV [17], [67]. Studies designed to obtain information on the genetic structure of IBDV from wild birds together with the use of the currently available phylogeographic tools may help bridge the gap regarding the emergence and spread of vvIBDV strains, as well as help elucidate the role of wild birds in the epidemiology of different IBDV strains.

The phylogeographic association-trait analysis revealed that viruses sampled from individual countries tend to cluster within them, suggesting a geographic subdivision among different strains of IBDV. On the other hand, some evidence of viral gene flow was also observed, suggesting the existence of epidemiological and commercial connections among different countries.

The most probable route of entry for the vvIBDV strains to Cuba was framed from the Netherlands, the United Kingdom and Belgium from 1993–1995. However, it is important to consider the fact that the virus circulating in Belgium was rapidly transmitted to the Netherlands. The origin in the United Kingdom appears to be the same as in Belgium and occurred in a very short period of time (see Video S1). Analysing the live poultry trade statistics of UN Comtrade, significant importations and exportations of birds among Belgium, the Netherlands and the United Kingdom occurred during 1987 (http://comtrade.un.org/db/). Therefore, the bird movements between these countries during this period of time could have favoured the emergence of viral populations of vvIBDV with few differences among them.

The importation of chicken to Cuba from the United Kingdom in the second half of the 1990’s (Dr. Fernández, A. Personal communication) is an important link between the chicken populations of both countries. Nonetheless, there was also an importation of chickens to Cuba from the Netherlands in 1993 (http://www.ceec.uh.cu/sites/default/…/LIBRO_CASOS_DE_ESTUDIO.pdf). Therefore, this last event seems to be the most probable cause of the introduction of vvIBDV strains to Cuban chicken flocks, even though multiple entries of the virus could have occurred at the same time.

The homogenous spread of the vvIBDV strains across the country could have been caused by the fact that the replacement of productive birds takes place for the entire country from the same genetic crossing from a single farm. Therefore, geographic divisions of the viral population have not been possible. Nevertheless, the evolutionary rate of the HVR-VP2 sequences from the Cuban viral population was estimated on the order of 10^−3^ nucleotide substitutions per site per year, which would be expected for RNA viruses (reviewed in [68], [69]). This value is slightly higher than the evolutionary rate estimated for VP2 sequences of the Brazilian viral population [65]. However, these differences could be explained because Silva et al. [65] used the whole VP2 sequence for their analyses, whereas only HVR-VP2 sequences region were analysed in the current work.

The demographic history of vvIBDV in Cuba showed a trend toward an initial growth of genetic diversity, possibly generated by the initial introduction of these strains. However, after the introduction of the CT-Gumboral vaccine in 1996, a decrease in the genetic diversity of the Cuban vvIBDV population was maintained (Dr. Alba, N. personal communication). This behaviour was further accentuated after the vaccination program supported by the use of the Cuban-produced vaccine, “Gumboro Vaccine” Labiofam, S.A., together with serological tests intended to determine the ideal time for vaccination. The decrease in genetic diversity observed for the Cuban vvIBDV population suggests that the disease was controlled in the country, even though eradication of this viral agent has not been accomplished. Nevertheless, the vaccination program has apparently been successful because it has maintained control of the new emerging strains. These latter strains were apparently linked to fixing the 299N mutation.

The replacement 299NxS seems to be the result of an antigenic drift event generated at random, but with an adaptive advantage, as has been suggested by the neutral hypothesis of the evolution (reviewed in [70]). This advantage could be caused by the closer distance between HVR-VP2 and the β-hairpin AA′ structures when an N residue was located in the 299 position.

The β-hairpin AA′ structure is an important stabilising element of the VP2 trimer during viral capsid formation [62]. This structure makes a flap that invades and completes the β-barrel of the neighbouring P domain in the trimer, contributing a fifth antiparallel strand at the edge of each β-sheet, making it more stable [62]. However, further studies are required to determine if the increased stabilisation of the VP2 trimer during viral capsid formation caused by the replacement 299NxS could have some impact in either the speed of viral replication or in viral pathogenesis.

In the current study, several codons of the HVR-VP2 analysed were found to be under purifying selection. However, these codons were mainly localised on the β-sheet. The β-sheet structures seem to be subjected to restrictions because they play an important role in the stability of the VP2 homotrimer, the unit that forms the viral capsid [62]. Thus, amino acid substitution in these structures could be deleterious for viral progeny.

### Conclusion

In this study, a rigorous measurement of the global phylogeographic dynamic of IBDV strains was performed based on HVR-VP2 sequences. This study revealed the expansion of strains carrying the sequences linked to the increased virulence of IBDV strains that appeared in Iran in 1981 and initially spread to Europe, Africa and East Asia, then later to the Caribbean Region and South America.

Framed in the Caribbean Region and Cuba, the present work is the first study that provides evidence that vvIBDV strains are circulating into Cuban poultry. The spatial and temporal analyses suggested that these strains were introduced to Cuba by an importation during the 1990’s, and once the virus arrived, it spread across the country. Nevertheless, this viral agent is under control by a successful vaccination program.

## Supporting Information

Figure S1 **Global flyways for migratory birds.**
(DOC)

Table S1 **IBDV sequences downloaded from the GenBank database used for different analyses in the current study.**
(DOC)

Table S2 **Coalescent priors and clock models compared by log marginal likelihood and AICM.** The best model was highlighted in boldface. All comparisons were based on equal numbers of independent Monte Carlo samples. SC = strict clock, UCDL = uncorrelated log-normal, UCDE = uncorrelated exponential. Const = constant population size, Exp = exponentially growing population size, Log = Logistic growing population size, BSP = Bayesian skyline plot.
(DOC)

Table S3 **Positive selection pressure analysis for HVR-VP2 sequence parameters estimated by the CODEML program implemented in the PAML package.**
(DOC)

Table S4 **Codons classified under negative selection pressure that were selected by SLAC with p<0.05.**
(DOC)

## References

[1] Liu HJ, Huang PH, Wu YH, Lin MY, Liao MH (2001) Molecular characterisation of very virulent infectious bursal disease viruses in Taiwan. Res Vet Sci 70: 139–147. 11356093 10.1053/rvsc.2001.0450

[2] Cosgrove AS (1962) An apparently new disease of chickens-avian nephrosis Avian Dis. 6: 385–389.

[3] Lasher HN, Davis VS (1997) History of infectious bursal disease in the USA-the first two decades Avian Dis. 41: 11–19. 9087316

[4] Dobos P, Hill BJ, Hallett R, Kells DT, Becht H, et al (1979) Biophysical and biochemical characterization of five animal viruses with bisegmented double-stranded RNA genomes. J Virol 32: 593–605. 228080 10.1128/jvi.32.2.593-605.1979 PMC353591

[5] Kibenge FSB, Dhillon AS, Rusell RG (1988) Biochemistry and immunology of infectious bursal disease virus. J Gen Virol 69: 1757–1775. 2841403 10.1099/0022-1317-69-8-1757

[6] Vakharia VN, He J, Ahamed B, Snyder DB (1994) Molecular basis of antigenic variation in infectious bursal disease virus. Virus Res 31: 265–273. 8178574 10.1016/0168-1702(94)90009-4

[7] Lukert PD, Saif YM (1997) Infectious bursal disease In: Calnek BW, Barnes HJ, Beard CW, McDougald LR, Saif YM editors. Diseases of Poultry 10th ed Iowa State University Press Ames IO 721–738.

[8] Rodríguez-Lecompte JC, Niño-Fong R, Lopez A, Frederick-Markham RJ, Kibenge FS (2005) Infectious bursal disease virus (IBDV) induces apoptosis in chicken B cells. Comp Immunol Microbiol Infect Dis 28: 321–337. 16188316 10.1016/j.cimid.2005.08.004

[9] McFerran JB, McNulty MS, McKillop ER, Connor TJ, McCracken RM, et al (1980) Isolation and serological studies with infectious bursal disease viruses from fowl turkeys and ducks: demonstration of a second serotype. Avian Pathol 9: 395–404. 18770277 10.1080/03079458008418423

[10] Ismail N, Saif YM, Moorhead PD (1988) Lack of pathogenicity of five serotype 2 infectious bursal disease viruses in chickens. Avian Dis 32: 757–759. 2849404

[11] Ismail N, Saif YM (1991) Immunogenicity of infectious bursal disease viruses in chickens. Avian Dis 35: 460–469. 1659364

[12] Van den Berg TP, Morales D, Enterradossi N, Rivallan G, Toquin D, et al (2004) Assessment of genetic antigenic and pathotypic criteria for the characterization of IBDV strains. Avian Pathol 1: 1–2. 10.1080/03079450400003650 15545026

[13] Chettle N, Stuart JC, Wyeth PJ (1989) Outbreak of virulent infectious bursal disease in East Anglia. Vet Rec 125: 271–272. 2552640 10.1136/vr.125.10.271

[14] Van den Berg TP, Meulemans G (1991) Acute infectious bursal disease in poultry; protection afforded by maternally derived antibodies and interference with live vaccination. Avian Pathol 20: 409–421. 18680037 10.1080/03079459108418779

[15] Jackwood DJ, Sommer-Wagner S (2007) Genetic characteristics of infectious bursal disease viruses from four continents Virology. 365: 369–375. 10.1016/j.virol.2007.03.046 17488648

[16] Banda A, Villegas P (2004) Genetic characterization of very virulent infectious bursal disease viruses from Latin America Avian Dis. 48: 540–549. 10.1637/7157-12304R 15529976

[17] Jeon WJ, Lee EK, Joh SJ, Kwon JH, Yang CB, et al (2008) Very virulent infectious bursal disease virus isolated from wild birds in Korea: Epidemiological implications. Virus Res 137: 153–156. 18652855 10.1016/j.virusres.2008.06.013

[18] Venereo M, Fonseca C, Espinosa M (1982) Primer reporte de Bursitis Infecciosa Aviar (enfermedad de Gumboro) en Cuba: First report of infectious bursal diseases virus in Cuba. Rev Cubana de Ciencias Veterinarias 13: 29–42.

[19] Fernández A, Bacallao A (1998) Situación actual de la Enfermedad de Gumboro en Cuba: Current situation of infectious bursal diseases virus in Cuba. Taller Internacional de Gumboro’98 Cuba: International Workshops of IBD 1998: 17–19.

[20] Perera CL, Noda J, Cuello S, Alfonso P, Espinosa V, et al.. (2005) Serology assisted vaccination against the infectious bursal diseases. REDVET 6:http://wwwveterinariaorg/revistas/redvet/n050505html. Accessed 2012 December 1.

[21] Babaahmady E, Joa R, Noda J (2005) Gumboro Disease Histopathology of the Bursa of Fabricio in the natural and experimental disease in fattening chicks. REDVET 4:http://wwwveterinariaorg/revistas/redvet/n040405html. Accessed 2012 December 1.

[22] González-Insua R, Silveira-Prado E, Olazábal-Manso E (2005) Frequency and characterization of anatomo-pathological lesions in gumboro disease and secondary illnesses associated under our environmental conditions A retrospective study. REDVET 4: http://wwwveterinariaorg/revistas/redvet/n101005html. Accessed 2012 December 1.

[23] Pybus OG, Perrins CM, Choudhury B, Manvell RJ, Núñez A, et al (2012) The ecology and age structure of a highly pathogenic avian influenza virus outbreak in wild mute swans Parasitology. 139: 1914–1923. 10.1017/S0031182012000261 22339986

[24] Faria NR, Suchard MA, Rambaut A, Lemey P (2011) Toward a quantitative understanding of viral phylogeography. Curr Opin Virol 1: 423–429. 22440846 10.1016/j.coviro.2011.10.003 PMC3312803

[25] de Carvalho LMF, Santos LBL, Faria NR, de Castro Silveira W (2013) Phylogeography of foot-and-mouth disease virus serotype O in Ecuador. Infect Gen and Evol 13: 76–88. 10.1016/j.meegid.2012.08.016 22985683

[26] Esbjörnsson J, Mild M, Månsson F, Norrgren H, Medstrand P (2011) HIV-1 Molecular Epidemiology in Guinea-Bissau, West Africa: Origin, Demography and Migrations. PLoS ONE 6: e17025. 21365013 10.1371/journal.pone.0017025 PMC3041826

[27] Perera CL, Noda J, Alfonso P, Cuello S, Rodríguez LM, et al (2002) Empleo de la inmunohistoquímica en el diagnóstico de la enfermedad infecciosa de la bolsa. Rev Cubana de Ciencia Avícola 26: 137–140.

[28] Moscoso H, Alvarado I, Hofacre CL (2006) Molecular Analysis of Infectious Bursal Disease Virus from Bursal Tissues Collected on FTA® Filter Paper. Avian Dis 50: 391–396. 17039839 10.1637/7505-011306R.1

[29] Noda J, Perera CL, Alfonso P, Cuello S, Espinosa V, et al (2003) Características patógenicas de algunos aislados del virus de la enfermedad infecciosa de la bolsa. Rev Cubana de Ciencias Avícolas 27: 139–145.

[30] Dolz R, Majó N, Ordóñez G, Porta R (2005) Viral Genotyping of Infectious Bursal Disease Viruses Isolated from the 2002 Acute Outbreak in Spain and Comparison with Previous Isolates. Avian Dis 49: 332–339. 16252484 10.1637/7299-110204R1.1

[31] Hall TA (1999) BioEdit: a user-friendly biological sequence alignment editor and analysis program for Windows 95/98/NT. Nucl Acids Symp Ser 4: 95–98.

[32] Padidam M, Sawyer S, Fauquet CM (1999) Possible emergence of new geminiviruses by frequent recombination. Virology 265: 218–225. 10600594 10.1006/viro.1999.0056

[33] Martin D, Rybicki E (2000) RDP: detection of recombination amongst aligned sequences Bioinformatics. 16: 562–563. 10.1093/bioinformatics/16.6.562 10980155

[34] Maynard-Smith J (1992) Analyzing the mosaic structure of genes. J Mol Evol 34: 126–129. 1556748 10.1007/BF00182389

[35] Posada D, Crandall KA (2001) Evaluation of methods for detecting recombination from DNA sequences: computer simulations. Proc Natl Acad Sci USA 98: 13757–13762. 11717435 10.1073/pnas.241370698 PMC61114

[36] Martin DP, Posada D, Crandall KA, Williamson C (2005) A modified BOOTSCAN algorithm for automated identification of recombinant sequences and recombination breakpoints. AIDS Res Hum Retroviruses 21: 98–102. 15665649 10.1089/aid.2005.21.98

[37] Gibbs MJ, Armstrong JS, Gibbs AJ (2000) Sister-scanning: a Monte Carlo procedure for assessing signals in recombinant sequences. Bioinformatics 16: 573–582. 11038328 10.1093/bioinformatics/16.7.573

[38] Boni MF, Posada D, Feldman MW (2007) An exact nonparametric method for inferring mosaic structure in sequence triplets. Genetics 176: 1035–1047. 17409078 10.1534/genetics.106.068874 PMC1894573

[39] Holmes EC, Worobey M, Rambaut A (1999) Phylogenetic evidence for recombination in dengue virus. Mol Biol Evol 16: 405–409. 10331266 10.1093/oxfordjournals.molbev.a026121

[40] Heath L, van der Walt E, Varsani A, Martin DP (2006) Recombination patterns in aphthoviruses mirror those found in other picornaviruses. J Virol 80: 11827–11832. 16971423 10.1128/JVI.01100-06 PMC1642601

[41] Posada D, Crandall K (1998) ModelTest: testing the model of DNA substitution Bioinformatics. 14: 817–818. 10.1093/bioinformatics/14.9.817 9918953

[42] Huelsenbeck JH, Ronquist F, Nielsen R, Bollback J (2001) Bayesian inference of phylogeny and its impact on evolutionary biology. Science 294: 2310–2314. 11743192 10.1126/science.1065889

[43] Ronquist F, Huelsenbeck JP (2003) MRBAYES 3: Bayesian phylogenetic inference under mixed models Bioinformatics. 19: 1572–1574. 10.1093/bioinformatics/btg180 12912839

[44] Guindon S, Gascuel O (2003) A simple fast and accurate algorithm to estimate large phylogenies by maximum likelihood. System Biol 52: 696–704. 14530136 10.1080/10635150390235520

[45] Kishino H, Hasegawa M (1989) Evaluation of the maximum likelihood estimate of the evolutionary tree topologies from DNA sequence data and the branching order in Hominoidea. J Mol Evol 29: 170–179. 2509717 10.1007/BF02100115

[46] Shimodaira H, Hasegawa M (1999) Multiple comparisons of log-likelihoods with applications to phylogenetic inference. Mol Biol Evol 16: 1114–1116.

[47] Yang Z, Wong WSW, Nielsen R (2005) Bayes empirical Bayes inference of amino acids sites under positive selection. Mol Biol Evol 22: 1107–1118. 15689528 10.1093/molbev/msi097

[48] Kishino H, Miyata T, Hasegawa M (1990) Maximum likelihood inference of protein phylogeny and the origin of chloroplasts. J Mol Evol 30: 151–160.

[49] Rambaut A (2008) FigTree v112 http://treebioedacuk/software/figtree.

[50] Drummond AJ, Suchard MA, Xie D, Rambaut A (2012) Bayesian phylogenetics with BEAUti and the BEAST 1.7. Mol Biol Evol 29: 1969–1973. 22367748 10.1093/molbev/mss075 PMC3408070

[51] Baele G, Lemey P, Bedford T, Rambaut A, Suchard MA, et al (2012) Improving the accuracy of demographic and molecular clock model comparison while accommodating phylogenetic uncertainty. Mol Biol Evol 29: 2157–2167. 22403239 10.1093/molbev/mss084 PMC3424409

[52] Drummond AJ, Rambaut A (2007) BEAST: Bayesian evolutionary analysis by sampling trees. BMC Evol Biol 7: 214. 17996036 10.1186/1471-2148-7-214 PMC2247476

[53] Parker J, Rambaut A, Pybus OG (2008) Correlating viral phenotypes with phylogeny: accounting for phylogenetic uncertainty. Infect Genet Evol 8: 239–246. 17921073 10.1016/j.meegid.2007.08.001

[54] Lemey P, Rambaut A, Drummond AJ, Suchard MA (2009) Bayesian phylogeography finds its roots. PLoS Comput Biol 5: e1000520. 19779555 10.1371/journal.pcbi.1000520 PMC2740835

[55] Bielejec F, Rambaut A, Suchard MA, Lemey P (2011) SPREAD: spatial phylogenetic reconstruction of evolutionary dynamics. Bioinformatics 27: 2910–2912. 21911333 10.1093/bioinformatics/btr481 PMC3187652

[56] Xia X, Xie Z (2001) DAMBE: software package for data analysis in molecular biology and evolution. J Hered 92: 371–373. 11535656 10.1093/jhered/92.4.371

[57] Nei M, Gojobori T (1986) Simple methods for estimating the numbers of synonymous and nonsynonymous nucleotide substitutions. Mol Biol Evol 3: 418–426. 3444411 10.1093/oxfordjournals.molbev.a040410

[58] Delport W, Poon AF, Frost SDW, Kosakovsky-Pond SL (2010) Datamonkey 2010: a suite of phylogenetic analysis tools for evolutionary biology. Bioinformatics 26: 2455–2457. 20671151 10.1093/bioinformatics/btq429 PMC2944195

[59] Kosakovsky-Pond SL, Frost SDW (2005) Datamonkey: rapid detection of selective pressure on individual sites of codon alignments. Bioinformatics 21: 2531–2533. 15713735 10.1093/bioinformatics/bti320

[60] Yang Z (2007) PAML 4:a program package for phylogenetic analysis by maximum likelihood. Mol Biol Evol 24: 1586–1591. 17483113 10.1093/molbev/msm088

[61] Anisimova M, Yang ZH (2007) Multiple hypothesis testing to detect lineages under positive selection that affects only a few sites. Mol Biol Evol 24: 1219–1228. 17339634 10.1093/molbev/msm042

[62] Coulibaly F, Chevalier C, Gutsche I, Pous J, Navaza J, et al (2005) The birnavirus crystal structure reveals structural relationships among icosahedral viruses. Cell 120: 761–772. 15797378 10.1016/j.cell.2005.01.009

[63] Hon CC, Lam TY, Drummond A, Rambaut A, Lee YF, et al (2006) Phylogenetic analysis reveals a correlation between the expansion of very virulent infectious bursal disease virus and reassortment of its genome segment B. J Virol. 80: 8503–8509. 10.1128/JVI.00585-06 PMC1563883 16912300

[64] Cortey M, Bertran K, Toskano J, Majó N, Dolz R (2012) Phylogeographic distribution of very virulent infectious bursal disease virus isolates in the Iberian Peninsula. Avian Pathol 41: 277–284. 22702455 10.1080/03079457.2012.682562

[65] Silva FM, Vidigal PM, Myrrha LW, Fietto JL, Silva A Jr, et al (2013) Tracking the molecular epidemiology of Brazilian Infectious bursal disease virus (IBDV) isolates. Infect Genet Evol 13: 18–26. 23000111 10.1016/j.meegid.2012.09.005

[66] Aghakhan SM, Abshar N, Fereidoni SR, Marunesi C, Khodashenas M (1994) Studies on avian viral infections in Iran. Archives of Razi Institute 44: 1–10.

[67] Kasanga CJ, Yamaguchi T, Wambura PN, Munangándu HM, Ohya K, et al (2008) Detection of infectious bursal disease virus (IBDV) genome in free-living pigeon and guinea fowl in Africa suggests involvement of wild birds in the epidemiology of IBDV. Virus Gen 36: 521–529. 10.1007/s11262-008-0219-z 18343984

[68] Domingo E, Escarmís C, Sevilla N, Moya A, Elena SF, et al (1996) Basic concepts in RNA virus evolution. FASEB J 10: 859–864. 8666162 10.1096/fasebj.10.8.8666162

[69] Elena SF, Miralles R, Moya A (1997) Frequency-dependent selection in a mammalian RNA virus. Evolution 51: 984–987. 28568603 10.1111/j.1558-5646.1997.tb03679.x

[70] Duret L (2008) Neutral theory: The null hypothesis of molecular evolution. Nature Education 1: 1.

