# Supplementary material for: Spatiotemporal Phylogenetic Analysis and Molecular Characterisation of Infectious Bursal Disease Viruses Based on the VP2 Hyper-Variable Region
Source: PLoS One. 2013 Jun 21;8(6):e65999. doi: 10.1371/journal.pone.0065999 (PMC3689766; doi:10.1371/journal.pone.0065999)
Supplement: Figure S1 — Global flyways for migratory birds. (DOC) [file pone.0065999.s001.doc]

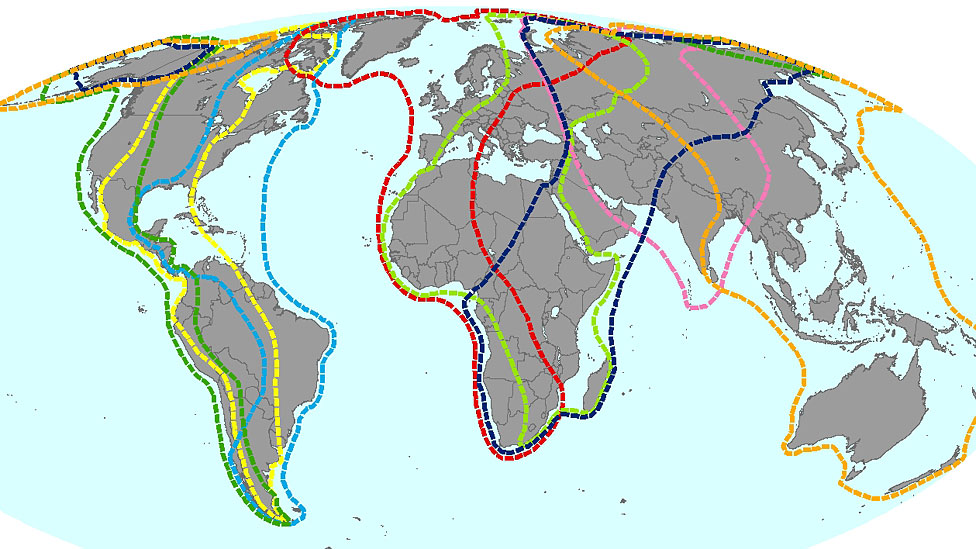
**Supplementary Fig. S1.** Global flyways for migratory birds

(Taken from: http://2.bp.blogspot.com/-jLKDUE1YvkA/TdEyCRGatlI/AAAAAAAACHw/0w9M87DVuRI/s1600/110513140714_global-flyways.jpg)
