# Supplementary material for: Spatiotemporal Phylogenetic Analysis and Molecular Characterisation of Infectious Bursal Disease Viruses Based on the VP2 Hyper-Variable Region
Source: PLoS One. 2013 Jun 21;8(6):e65999. doi: 10.1371/journal.pone.0065999 (PMC3689766; doi:10.1371/journal.pone.0065999)
Supplement: Table S1 — IBDV sequences downloaded from the GenBank database used for different analyses in the current study. (DOC) [file pone.0065999.s002.doc]

| **ID used in tree** | **strain** | **year of collection** | **country** | **GenBank No.** |
| --- | --- | --- | --- | --- |
| X92760@89_UK | UK661 | 1989 | United Kingdom | X92760 |
| D49706@91_JP | OKJM | 1991 | Japan | D49706 |
| AF092943@94_HK | HK46 | 1994 | Hong Kong | AF092943 |
| D16630@88_NL | DV86 | 1988 | Netherlands | D16630 |
| AF533670@92_CN | SH/92 | 1992 | China | AF533670 |
| AY321949@87_BE | 849VB | 1987 | Belgium | AY321949 |
| AY770581@02_ES | SP/03/02 | 2002 | Spain | AY770581 |
| AF159218@89_EG | K406/89 | 1989 | Egypt | AF159218 |
| AB024076@91_JP | Ehime91 | 1991 | Japan | AB024076 |
| JF682254@05_ES | GAL222-05 | 2005 | Spain | JF682254 |
| JF682292@09_ES | RIO119-09 | 2009 | Spain | JF682292 |
| JF682255@05_ES | GAL224-05 | 2005 | Spain | JF682255 |
| JF682297@09_ES | GAL141-09 | 2009 | Spain | JF682297 |
| EU835867@99_BR | Br/99/BN | 1999 | Brazil | EU835867 |
| JF811919@01_BR | MG-7 | 2001 | Brazil | JF811919 |
| EU835873@03_BR | Br/03/DT | 2003 | Brazil | EU835873 |
| EU835874@03_BR | Br/03/DU | 2003 | Brazil | EU835874 |
| DQ630451@82_IR | RT75D/82 | 1982 | Iran | DQ630451 |
| DQ630455@81_IR | RT275/81 | 1981 | Iran | DQ630455 |
| D00869@70_UK | F 52/7 | 1970 | United Kingdom | D00869 |
| D00499@67_USA | STC | 1967 | United States | D00499 |
| Y14962@78_NL | D-78 | 1978 | Netherlands | Y14962 |
| D00867@75_DE | Cu1wt | 1975 | Germany | D00867 |
| AY770585@02_ES | SP/14/02 | 2002 | Spain | AY770585 |
| GQ866120@09_TW | P98/02 | 2009 | Taiwan | GQ866120 |
| GU299811@09_TW | P98/21 | 2009 | Taiwan | GU299811 |
| AY311479@01_EG | Kal2001 | 2001 | Egypt | AY311479 |
| GU299810@09_TW | P98/20 | 2009 | Taiwan | GU299810 |
| M97346@87_USA | GLS | 1987 | United States | M97346 |
| M64285@85_USA | Del-A | 1985 | United States | M64285 |
| X54858@85_USA | Del-E | 1985 | United States | X54858 |
| AF148081@95_AU | 08/95 | 1995 | Australia | AF148081 |
| AF148076@94_AU | 01/94 | 1994 | Australia | AF148076 |
| AJ508758@00_PL | 00/40 | 2000 | Poland | AJ508758 |
| AY321950@96_FR | 96108 | 1996 | France | AY321950 |
| JF682279@08_ES | NAV783-08 | 2008 | Spain | JF682279 |
| AJ878902@99_BR | 99009 | 1999 | Brazil | AJ878902 |
| AF240686@89_NL | D6848 | 1989 | Netherlands | AF240686 |
| AJ001944@91_FR | 91247 | 1991 | France | AJ001944 |

**Table S1.** IBDV sequences downloaded from the GenBank database used for different analyses in the current study.

**Table S1. Cont.**

|  | **ID used in tree** | **strain** | **year of collection** | **country** | **GenBank No.** |
| --- | --- | --- | --- | --- | --- |
|  | Y14956@89_FR | 89163 | 1989 | France | Y14956 |
|  | AY029166@67_USA | IM | 1967 | United States | AY029166 |
|  | AY918950@67_USA | Edgar | 1967 | United States | AY918950 |
|  | D00868@76_UK | PBG-98 | 1976 | United Kingdom | D00868 |
|  | M66722@82_USA | OH | 1982 | United States | M66722 |
| **‡** | EF138983_CA | 03-22204 | na | Canada | EF138983 |
| **‡** | EF418034_USA | GA-1 | na | United States | EF418034 |
| **‡** | AF533672_KR | 225V4 | na | Korea | AF533672 |
| **‡** | AJ310185_FR | Strain CT | na | France | AJ310185 |
| **‡** | AF281232_USA | Bursine | na | United States | AF281232 |
| **‡** | DQ202329_CN | B87 | na | China | DQ202329 |
| **‡** | EF138988_CA | 03-27950-dn | na | Canada | EF138988 |
| **‡** | AF312371_CN | T2 | na | China | AF312371 |
| **‡** | AF533676_KR | 269V4 | na | Korea | AF533676 |
| **‡** | AF533678_KR | K1V4 | na | Korea | AF533678 |
| **‡** | AY769978_BR | Ipumirim-BR | na | Brazil | AY769978 |
| **‡** | EU082025_IR | IR299 | na | Iran | EU082025 |
| **‡** | DQ825652_CN | HZ | na | China | DQ825652 |
| **‡** | AY769979_BR | Suruvi-BR | na | Brazil | AY769979 |
| **‡** | AY780418_BR | SM-BR | na | Brazil | AY780418 |
| **‡** | EU091537_IR | IR599 | na | Iran | EU091537 |
| **‡** | AY780423_BR | JNeto-BR | na | Brazil | AY780423 |
| **‡** | EU417823_CN | AH1 | na | China | EU417823 |
| **‡** | AY628217_CN | Y3 | na | China | AY628217 |
| **‡** | EU328333_CN | SH-h | na | China | EU328333 |
| **‡** | AY628215_CN | P8G | na | China | AY628215 |
| **‡** | AY772164_IR | IR398 | na | Iran | AY772164 |
| **‡** | EU091535_IR | IR399 | na | Iran | EU091535 |

na: not available

**‡**: sequences removed for inference of date Bayesian analysis and discrete phylogeographic analysis
