# Supplementary material for: Spatiotemporal Phylogenetic Analysis and Molecular Characterisation of Infectious Bursal Disease Viruses Based on the VP2 Hyper-Variable Region
Source: PLoS One. 2013 Jun 21;8(6):e65999. doi: 10.1371/journal.pone.0065999 (PMC3689766; doi:10.1371/journal.pone.0065999)
Supplement: Table S2 — Coalescent priors and clock models compared by log marginal likelihood and AICM. The best model was highlighted in boldface. All comparisons were based on equal numbers of independent Monte Carlo samples. SC = strict clock, UCDL = uncorrelated log-normal, UCDE = uncorrelated exponential. Const = constant population size, Exp = exponentially growing population size, Log = Logistic growing population size, BSP = Bayesian skyline plot. (DOC) [file pone.0065999.s003.doc]

# Table S2. Coalescent priors and clock models compared by log marginal likelihood and AICM. The best model was highlighted in boldface. All comparisons were based on equal numbers of independent Monte Carlo samples. SC=strict clock, UCDL=uncorrelated log-normal, UCDE=uncorrelated exponential. Const=constant population size, Exp=exponentially growing population size, Log=Logistic growing population size, BSP=Bayesian skyline plot.

| **Clock Model** | **Coalescent** | **Log-likelihood** | **ESS** | **Marginal Likelihood (S.E.)** | **Log-Marginal Likelihood (Harmonic Mean)** | **Log-Marginal Likelihood (smoothed Harmonic Mean)** | **AICM** |
| --- | --- | --- | --- | --- | --- | --- | --- |
| SC | Const | -2269.3323 | 5513.5336 | -2287.292 (0.108) | -2305.9911 | -2287.3189 | 4692.2234 |
| SC | Exp | -2268.8365 | 4447.4216 | -2286.187 (0.113) | -2303.2577 | -2286.2891 | 4684.3736 |
| SC | Log | -2289.6424 | 7098.2815 | -2308.384 (0.114) | -2335.0363 | -2308.3037 | 4747.1543 |
| SC | BSP | -2264.8627 | 3733.3321 | -2281.863 (0.112) | -2297.6624 | -2281.7593 | 4666.2955 |
| UCDE | Const | -2238.864 | 1550.209 | -2260.163 (0.136) | -2276.2799 | -2260.0755 | 4685.1879 |
| UCDE | Exp | -2238.91 | 1453.598 | -2260.024 (0.136) | -2282.6975 | -2259.964 | 4684.0941 |
| UCDE | Log | -2249.07 | 907.061 | -2270.931 (0.131) | -2290.7039 | -2270.8115 | 4717.7124 |
| **UCDE** | **BSP** | **-2236.0959** | **1459.2082** | **-2256.497 (0.128)** | **-2274.3349** | **-2256.5488** | **4666.1790** |
| UCDL | Const | -2236.0987 | 1184.5031 | -2257.901 (0.142) | -2350.0482 | -2258.4333 | 4704.6369 |
| UCDL | Exp | -2236.6 | 1213.0618 | -2258.646 (0.141) | -2279.2427 | -2258.8081 | 4698.4484 |
| UCDL | Log | -2235.8982 | 607.587 | -2257.693 (0.137) | -2412.3325 | -2258.5822 | 4746.6297 |
| UCDL | BSP | -2238.2403 | 269.5248 | -2262.01 (0.149) | -2330.7400 | -2262.2195 | 4731.7824 |
