# Supplementary material for: Spatiotemporal Phylogenetic Analysis and Molecular Characterisation of Infectious Bursal Disease Viruses Based on the VP2 Hyper-Variable Region
Source: PLoS One. 2013 Jun 21;8(6):e65999. doi: 10.1371/journal.pone.0065999 (PMC3689766; doi:10.1371/journal.pone.0065999)
Supplement: Table S3 — Positive selection pressure analysis for HVR-VP2 sequence parameters estimated by the CODEML program implemented in the PAML package. (DOC) [file pone.0065999.s004.doc]

**Table S3. Positive selection pressure analysis for HVR-VP2 sequence parameters estimated by the CODEML program implemented in the PAML package.**

| **Region** | **Model** | ***Log-likelihood scores*** | **Parameters** |  |  |  |  | **Positively selected sites p<0.05** |
| --- | --- | --- | --- | --- | --- | --- | --- | --- |
| HVR-VP2 | M0 | -2060.16 | ω= 0.16383 |  |  |  |  | - |
|  | M1 | -2050.59 | p0= 0.87544 | p1=0.12456 |  |  |  | - |
|  | M2 | -2050.59 | ω= 1.877 | p0=0.87544 | p1=1.00000 | p2=0.000 |  | - |
|  | M7 | -2048.49 | p= 0.54254 | q= 2.46639 |  |  |  | - |
|  | M8 | -2047.58 | ω= 1.63566 | p0= 0.99240 | p= 0.65084 | q= 3.26349 | (p1= 0.00760) | - |
