# Supplementary material for: Spatiotemporal Phylogenetic Analysis and Molecular Characterisation of Infectious Bursal Disease Viruses Based on the VP2 Hyper-Variable Region
Source: PLoS One. 2013 Jun 21;8(6):e65999. doi: 10.1371/journal.pone.0065999 (PMC3689766; doi:10.1371/journal.pone.0065999)
Supplement: Table S4 — Codons classified under negative selection pressure that were selected by SLAC with p<0.05. (DOC) [file pone.0065999.s005.doc]

**Table S4. Codons classified under negative selection pressure that were selected by SLAC with p<0.05**

| **Codon position** | **dN-dS** | **p-value** |
| --- | --- | --- |
| 207 | -3.0477 | 0.037037 |
| 210 | -3.0477 | 0.037037 |
| 224 | -4.0000 | 0.012592 |
| 225 | -3.0000 | 0.037037 |
| 232 | -4.0000 | 0.012346 |
| 234 | -3.2684 | 0.028648 |
| 236 | -4.0000 | 0.012346 |
| 239 | -3.2926 | 0.041049 |
| 248 | -3.2926 | 0.041049 |
| 258 | -4.5515 | 0.019836 |
| 276 | -3.0000 | 0.037037 |
| 285 | -3.0000 | 0.037057 |
| 288 | -4.9389 | 0.008317 |
| 293 | -3.2926 | 0.041049 |
| 298 | -3.0000 | 0.037037 |
| 313 | -3.0000 | 0.037037 |
| 319 | -4.0000 | 0.012358 |
| 321 | -3.5256 | 0.044334 |
| 338 | -4.9389 | 0.044334 |
| 339 | -4.0000 | 0.012355 |
| 345 | -3.0477 | 0.037037 |
| 350 | -4.0635 | 0.012346 |
